# Physical Activity, Sunshine Duration, and Osteoporotic Fractures: A Nested Case-Control Study

**DOI:** 10.3390/jpm12020164

**Published:** 2022-01-26

**Authors:** Chanyang Min, Dae-Myoung Yoo, Mi-Jung Kwon, Joo-Hee Kim, Hyo-Geun Choi

**Affiliations:** 1 Hallym Data Science Laboratory, Hallym University College of Medicine, Anyang 14068, Korea; joicemin@naver.com (C.M.); ydm1285@naver.com (D.-M.Y.); 2 Department of Pathology, Hallym Sacred Heart Hospital, Hallym University College of Medicine, Anyang 14068, Korea; mulank@hanmail.net; 3 Division of Pulmonary, Allergy and Critical Care Medicine, Department of Medicine, Hallym Sacred Heart Hospital, Hallym University College of Medicine, Anyang 14068, Korea; luxjhee@gmail.com; 4 Department of Otorhinolaryngology—Head and Neck Surgery, Hallym University College of Medicine, Anyang 14068, Korea

**Keywords:** physical activity, sunshine duration, osteoporotic fractures, vertebral fracture, hip fracture, distal radius fracture

## Abstract

This study examined the associations between the occurrence of osteoporotic fractures in detailed sites and combined physical activity (PA) and sunshine duration (SD). Data from the Korean National Health Insurance Service—National Health Screening Cohort for 7-year periods and from the Korea Meteorological Administration were used. Osteoporotic fractures (*n* = 12,103), including vertebral fractures, hip fractures, and distal radius fractures, and matched controls (*n* = 24,206) were selected in 1:2 ratios by age, sex, income, and region of residence. PA was classified as moderate- to high-intensity PA (High PA) and low-intensity PA (Low PA). SD was classified as Short SD (<6.1 h) and Long SD (≥6.1 h). Conditional logistic regression was used to calculate the odds ratios (ORs) with 95%-confidence intervals (CIs) of the combined PA and SD groups for the occurrence of each osteoporotic fracture. Compared to ‘Low PA + Short SD’, the adjusted ORs (95% CIs) for vertebral fracture in ‘High PA + Short SD’ and ‘High PA + Long SD’ were 0.83 (0.76–0.91) and 0.84 (0.77–0.92), respectively. Hip/distal radius fractures were not associated with the combined PA and SD group. We suggest that a higher intensity of PA is inversely associated with the risk of vertebral fracture.

## 1. Introduction

Physical inactivity is one of the major health problems worldwide. For the COVID-19 pandemic, physical activity (PA) is recommended not only for severe COVID-19 infection itself [1], but also for physical and mental disorders [2]. However, indoor leisure grouped PA is restricted due to the possible spread of infection [3]. Instead, the trend for PA was to change to home-based personal PA or outdoor leisure PA [2].

Outdoor PA could increase sun exposure, which improves vitamin D status in the body. Both PA and sun exposure are effective in improving musculoskeletal conditions, especially increasing bone mineral density (BMD) [4].

Osteoporotic fractures caused by decreased BMD with falls could lead to death in elderly individuals. Because aging is ongoing worldwide, fracture is considered one of the major health problems [5]. The incidence of vertebral fracture rises 20-fold in women and 10-fold in men when individuals are 60 to 90 years old [6]. Hip fracture is the most severe osteoporotic fracture and is associated with 20% mortality and 50% functional loss [7]. In the United Kingdom, the annual incidences of distal radius fracture for >35-year-old males and females were 9 and 37 per 10,000, respectively [8].

Increasing evidence with large data from population studies demonstrated that PA could reduce the risk of osteoporotic fractures, especially hip fractures [9,10]. However, the findings of vertebral fracture were inconsistent depending on the studies [11,12,13], and distal radius fracture was not associated with PA [9,10]. Regarding vitamin D, some studies investigated the association between outdoor activity and fractures and suggested that more outdoor activity could reduce fractures [14,15]. One study suggested that a low prevalence of vertebral fractures was associated with increased lifetime ultraviolet (UV) radiation exposure using Beagley—Gibson (BG) grade in women [16]. However, few studies have investigated the association between specific sites of osteoporotic fractures and combined PA and sun exposure.

Our previous study, which used the same national cohort data and meteorological data as our present study, found that a long sunshine duration (SD) and higher intensity PA were associated with a lower occurrence of osteoporosis [17]. A study using national cohort data from Korea also reported that PA was associated with low fracture risk regardless of the type of PA and site of fracture [18].

Based on previous studies, we hypothesized that a higher intensity of PA with a long SD might be associated with a lower occurrence of each osteoporotic fracture. The purpose of our current study was to identify the association between osteoporotic fractures in specific sites (spine, hip, and wrist) and combined PA and SD using national cohort data.

## 2. Materials and Methods

### 2.1. Study Population and Participant Selection

This study was approved by the ethics committee of Hallym University (HALLYM 2019-08-029) following the guidelines of the Institutional Review Board (IRB).

The Korean National Health Insurance Service—National Health Screening Cohort (NHIS—HEALS) was used in this study. The details of the Korean NHIS—HEALS have been described elsewhere [19].

**Figure 1:**
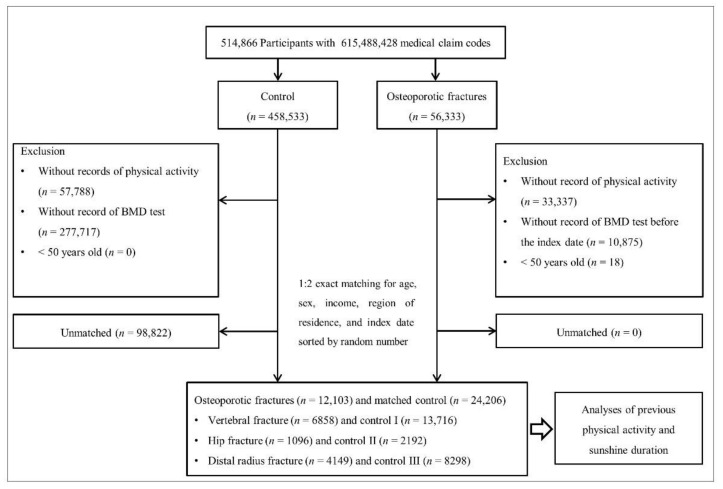
A schematic illustration of the participant selection process that was used in the present study. Of a total of 514,866 participants, 12,103 osteoporotic fractures were matched with 24,206 control participants for age, sex, income, and region of residence. BMD, bone mineral density.

Among 514,866 participants, 56,333 were included in the osteoporotic fracture group according to the definition of each fracture in our study, and 458,533 were included in the control group. Because the PA information was different from 2002 to 2008, those participants and participants without PA records were removed (*n* = 33,337 in the osteoporotic fractures group; *n* = 57,788 in the control group). In the osteoporotic fractures group, participants who did not undergo the BMD test before the first fracture diagnosis (index date) were excluded (*n* = 10,875; claim codes were described elsewhere [17]). In the control group, participants who did not undergo the BMD test from 2002 to 2015 were excluded (*n* = 277,717). The matching method that was previously used in our studies was conducted [17,20]. The total fracture group and control group were matched in 1:2 ratios by age, sex, income, and region of residence. Each control participant was assigned the same index date as each matched fracture participant. Control participants who did not have a BMD test before the index date were excluded from matching. During the matching process, 98,822 control participants were excluded. Finally, 12,103 osteoporotic fracture patients and 24,206 control patients were included in the analysis. Furthermore, the fractures and matched controls were classified into specific sites of fracture as follows: 6858 in the vertebral fracture group and 13,716 in the control I group; 1096 in the hip fracture group and 2192 in the control II group; 4149 in the distal radius fracture group and 8298 in the control III group. Each fracture group and matched control group were analyzed for previous PA and SD (Figure 1).

### 2.2. Definition of Osteoporotic Fractures (Outcome)

Osteoporotic fractures were vertebral fractures, hip fractures, and distal radius fractures. All fractures were defined using the codes of International Classification of Diseases 10th Edition (ICD-10). Vertebral fracture was defined if the participants were diagnosed with S220 (fracture of thoracic vertebra) or S320 (fracture of lumbar vertebra). Hip fracture was defined if the participants were diagnosed with S720 (fracture of neck of femur), S721 (pertrochanteric fracture), or S722 (subtrochanteric fracture). Distal radius fracture was defined if the participants were diagnosed with S525 (fracture of lower end of radius).

### 2.3. Classification of Physical Activity and Sunshine Duration (Exposure)

PA information was collected using a modified International Physical Activity Questionnaire (IPAQ) [21]. We used PA information from the first record of health screening. PA was classified as our previous study [17]. Specifically, ‘moderate- to high-intensity PA (High PA)’ was defined as walking ≥5 days in ≥30 min at a time, performing a moderate-intensity activity ≥5 days in ≥30 min at a time, performing a vigorous-intensity activity ≥3 days in ≥20 min at a time, or any combination of walking, moderate-intensity activity, or vigorous-intensity activity ≥5 days with ≥600 metabolic equivalent (MET)-min/week based on the IPAQ. Other participants were classified as ‘low-intensity PA (Low PA)’.

SD data were collected by the Korea Meteorological Administration (KMA), which takes measurements hourly using an automated synoptic observing system (ASOS) plus manual measurement at 94 locations [22]. We linked SD data and NHIS-HEALS data by residential area and index date. The residential areas were classified according to 16 areas categorized by NHIS-HEALS. The mean value of SD for 1 year (365 days) before the index date was calculated for each participant. SD was classified into ‘Short SD’ (<6.1 h) and ‘Long SD’ (≥6.1 h) based on the median value of SD (6.1 h).

PA and SD were combined into 4 groups as follows: ‘Low PA + Short SD’, ‘Low PA + Long SD’, ‘High PA + Short SD’, and ‘High PA + Long SD’.

### 2.4. Covariates

Age, sex, income, and region of residence were used as covariates in analyses and performed as described in our previous study [20,23]. Age was collected and classified from patients ≥50 years old at 5-year intervals. The income groups were classified from the 1 (low) to 5 (high) level, and region of residence was divided into urban and rural. Tobacco smoking status (nonsmoker, past smoker, or current smoker), alcohol consumption (<1 time a week or ≥1 time a week), and obesity based on BMI (<18.5 for underweight, ≥18.5 to <23 for normal weight, ≥23 to <25 for overweight, ≥25 to <30 for obese I, or ≥30 for obese II) were classified in the same way as elsewhere [20,23]. Osteoporosis was defined if participants were diagnosed with M80 (osteoporosis with pathological fracture), M81 (osteoporosis without pathological fracture), or M82 (osteoporosis in diseases classified elsewhere) using ICD-10 codes before the index date. To evaluate the burden of comorbidities, the Charlson Comorbidity Index (CCI) score was used [24].

### 2.5. Statistical Analyses

The general characteristics were compared between each type of fracture and matched control groups using the absolute standardized difference (sd) [25]. Conditional logistic regression was used to analyze the odds ratios (ORs) with 95% confidence intervals (CIs) for each type of fracture among the combined PA and SD groups. We used ‘Low PA + Short SD’ as reference group. In this analysis, crude and adjusted models were calculated. The adjusted model was adjusted for obesity, smoking status, alcohol consumption, osteoporosis, and CCI scores. The analysis was stratified by age, sex, income, and region of residence. We calculated the *p*-value for the interaction for each fracture between each variable and the combined PA and SD groups. For the subgroup analyses, we regrouped participants by combined age group (<60 years old and ≥60 years old) and sex and analyzed them using a conditional logistic regression model. Additionally, subgroup analyses according to osteoporosis history (nonosteoporosis vs. osteoporosis) was assessed using unconditional logistic regression.

Other subgroup analyses were performed according to covariates (Tables S1–S3).

Two-tailed analyses were performed, and significance was indicated by a *p*-value < 0.05. Since we used three types of fractures as dependent variables, Bonferroni correction was used to avoid type 1 error when calculating the *p*-value (α = 0.05/3). We used SAS version 9.4 (SAS Institute Inc., Cary, NC, USA) for statistical analyses.

## 3. Results

### 3.1. General Characteristics

The rate of high PA was relatively lower in the vertebral fracture group (37.1% (2547/6858) vs. 41.4% (5680/13,716); sd = 0.09) and in the hip fracture group (35.2% (386/1096) vs. 39.7% (870/2192); sd = 0.09) but not in the distal radius fracture group compared to each matched control group. The rate of long SD was relatively lower in the vertebral fracture group (48.8% (3344/6858) vs. 49.6% (6804/13,716); sd = 0.02) and in the distal radius fracture group (50.0% (2075/4149) vs. 50.3% (4175/8298); sd = 0.01) and higher in the hip fracture group (53.4% (585/1096) vs. 51.1% (1119/2192); sd = 0.05) than in each matched control group (Table 1).

### 3.2. Odds Ratios for Vertebral Fracture, Hip Fracture, and Distal Radius Fracture

Compared to ‘Low PA + Short SD’, the adjusted ORs (95% CIs) for vertebral fracture in ‘Low PA + Long SD’, ‘High PA + Short SD’, and ‘High PA + Long SD’ were 0.98 (0.90–1.05; *p* = 0.519), 0.83 (0.76–0.91; *p* < 0.001), and 0.84 (0.77–0.92; *p* < 0.001), respectively (Table 2). However, hip/distal radius fractures were not statistically significant in any combined PA and SD group (Table 3 and Table 4).

### 3.3. Subgroup Analyses of Odds Ratios for Vertebral Fracture

The findings from vertebral fracture were consistent with the subgroup of patients ≥60 years old and the subgroups of males and females. In the <60-year-old subgroup, ‘Low PA + Long SD’ had a statistically lower OR than ‘Low PA + Short SD’, as well as ‘High PA + Short SD’ and ‘High PA + Long SD’. In the osteoporosis subgroup, ‘High PA + Short SD’ had a significantly lower OR than ‘Low PA + Short SD’. In the nonosteoporosis subgroup, ‘High PA + Long SD’ had a statistically lower OR than ‘Low PA + Short SD’ (Table 2). 

## 4. Discussion

To our knowledge, our study is the first to investigate the occurrence of osteoporotic fractures depending on the combined PA and SD status using national cohort data and national meteorological data. We found that a lower occurrence of vertebral fracture was associated with ‘High PA + Short SD’ and ‘High PA + Long SD’. However, hip/distal radius fracture did not show an association.

Previous studies regarding the association between PA and osteoporotic fractures have been controversial depending on the specific site of fracture [26]. According to one meta-analysis using nine prospective cohort studies, PA was inversely associated with the risk of hip fracture (relative risk = 0.93; 95% CI = 0.91–0.96), and one review study also reported that PA could reduce the risk of hip fracture by 20–40%. However, an association between PA and distal radius fracture was not found in either study [9,10]. Regarding vertebral fracture, the findings were different depending on the study design and subjects. One RCT study with postmenopausal women reported that an additional risk of vertebral fracture was not found in the high-intensity resistance and impact training (HiRIT) group over 8 months. Instead, the improvement in the degree of thoracic kyphosis was significantly higher in the HiRIT group than in the low-intensity home-based exercise (CON) group (inclinometer-determined thoracic kyphosis: HiRIT group vs. CON group = −6.7 ± 8.2° vs. −1.6 ±8.1°; *p* = 0.031) [13]. One cohort study reported that ≥ four times a week of leisure-time PA (LTPA) in 31-year-old women resulted in a 47 cm^2^ (4.5%) larger vertebral cross-sectional area than the reference group [12]. However, an association between PA and vertebral fracture was not shown in one observational study. These results might have been due to the small number of participants (*n* < 2000) [11].

Several studies have investigated the association between sunlight exposure and vitamin D and fractures. One study from Sweden reported the correlation between latitude in Sweden and the risk of hip fracture, and the correlation between UV radiation and hip fracture. According to the study findings, the higher the latitude, the higher the risk of hip fracture in all age- and sex-specific groups (r = 0.191 to 0.308; *p* ≤ 0.001). Moreover, the greater UV radiation exposure, the lower the risk of hip fracture in all age- and sex-specific groups (r = −0.131 to −0.912; *p* ≤ 0.029) [27]. Another study reported that an increasing Beagley—Gibson grade, the measurement of cumulative lifetime UV radiation, was associated with a lower vertebral fracture prevalence in females (OR = 0.44; 95% CI = 0.22–0.87) [16]. Few studies have been conducted on the association between distal radius fracture and sun exposure.

Although we combined two variables as one, our finding that the association of low occurrence of vertebral fracture in ‘High PA + Short SD’ and ‘High PA + Long SD’ was consistent with the findings from previous studies [12,13,16]. Although PA alone was associated with vertebral fracture, SD alone was not associated with vertebral fracture in our study data (Table S4). In addition, ‘Low PA + Long SD’ was not associated with the main results in our study. In this regard, ‘High PA’ might be a crucial factor to prevent vertebral fracture regardless of whether a ‘Long SD’ exists. In another aspect, the SD might also be one of the indicators of outdoor PA because SD is one of the weather factors [28,29]. A previous study reported that the more time spent outdoors, the lower the risk of hip fracture. However, when the physical functioning score was adjusted in the model, the association disappeared [30]. Likewise, the findings of our study in the subgroup analysis according to osteoporosis history were similar to those of the previous study. In detail, for the nonosteoporosis subgroup, a low occurrence of vertebral fracture was shown only in the ‘High PA + Long SD’ subgroup, whereas for the osteoporosis subgroup, a low occurrence was shown only in the ‘High PA + Short SD’ subgroup. In other words, nonosteoporotic individuals could prevent vertebral fracture due to ‘High PA’ with a ‘Long SD’, whereas osteoporotic individuals could prevent vertebral fracture due to ‘High PA’ with a ‘Short SD’. Based on the previous study and our current findings, long SD might rather be a risk factor for increased fractures among individuals with impaired physical function.

On the other hand, only the <60-year-old subgroup had a low occurrence of vertebral fracture in ‘Low PA + Long SD’ in our study. In one previous case report, a 33-year-old man was reported to have bilateral femoral neck (hip) fracture with low T-scores in the lumbar spine and femoral neck. The authors discussed that the fracture and low T-score occurred because the man had not been exposed to sunlight for 3 years [31]. In other words, this report shows that even a healthy young man could have bone loss and fracture if he had no exposure to sunlight for a long period. In addition, the risk factor for fracture is higher in older age than in younger age [30]. Hence, increasing sun exposure at a relatively younger age may have beneficial effects in preventing vertebral fracture.

In our study, the number of vertebral fractures (*n* = 6858) was higher than that of other fractures (*n* = 1096 for hip fracture; *n* = 4149 for distal radius fracture). An association between hip/distal radius fracture and combined PA and SD might not have been found due to the smaller number of participants. Moreover, because SD is a broad indicator of vitamin D, the association might not be evident. In fact, PA was associated with a low occurrence of hip fracture in each normal weight subgroup, the less-than-once-a-week alcohol consumption subgroup, and the nonosteoporosis subgroup, which was consistent with previous studies (Table S5) [9,10]. SD alone was not associated with the occurrence of distal radius fractures or PA, which is consistent with previous studies (Table S6) [9,10]. Therefore, combined PA and SD might also not be associated with distal radius fracture.

The strengths of our study are as follows: Our study used large, national cohort data with a 7-year follow-up period. We used multiple variables as covariates, such as obesity, smoking status, alcohol consumption, and osteoporosis history, and used matching methods to determine whether osteoporotic fractures were independently associated with combined PA and SD history. We specified each osteoporotic fracture as a vertebral fracture, hip fracture, or distal radius fracture because each site of bone has unique characteristics. The KMA data and the national cohort data were merged so that SD was measured as a broad indicator of vitamin D. In participant exclusion, we excluded participants who lacked BMD data and therefore could not be categorized as either osteoporotic or nonosteoporotic.

One of the major limitations of our study is the use of secondary data with limited variables. For example, we could not consider traumatic events (e.g., falls), dietary vitamin D or calcium intake, or supplementation with vitamin D or calcium—factors which may modify the risk of fractures or affect the onset of BMD. PA groups were classified into only ‘Low PA’ and ‘High PA’ because the calculation of METs was limited. In addition, the type of PA was not specified because of limited information. The SD variable is another limitation in that it provides limited information regarding individuals’ actual sunlight exposure. In addition, although we used large cohort data from health insurance holders in Korea, the study findings should be carefully applied because the data do not represent the Korean population. Finally, due to this being an observational study, the determination of causality is not evident between combined PA and SD and osteoporotic fractures.

## 5. Conclusions

We suggest that ‘High PA’ is associated with a decreased risk of vertebral fracture regardless of SD. ‘Long SD’ could reduce the risk of vertebral fracture only in younger age as well as with ‘High PA’. Nonosteoporotic individuals are recommended to have a ‘High PA’ with ‘Long SD’, whereas osteoporotic individuals are recommended to have a ‘High PA’ with ‘Short SD’.

## Figures and Tables

**Table 1:** General characteristics of participants.

Characteristics	Vertebral Fracture and Matched Control			Hip Fracture and Matched Control			Distal Radius Fracture and Matched Control		
	Vertebral Fracture (n; %)	Control I (n; %)	Standardized Difference ^1^	Hip Fracture (n; %)	Control II (n; %)	Standardized Difference	Distal Radius Fracture (n; %)	Control III (n; %)	Standardized Difference
***n***	6858	13,716		1096	2192		4149	8298	
**Age (years old)**			<0.001			<0.001			<0.001
50–54	110 (1.6)	220 (1.6)		15 (1.4)	30 (1.4)		275 (6.6)	550 (6.6)	
55–59	328 (4.8)	656 (4.8)		28 (2.6)	56 (2.6)		681 (16.4)	1362 (16.4)	
60–64	680 (9.9)	1360 (9.9)		60 (5.5)	120 (5.5)		778 (18.8)	1556 (18.8)	
65–69	982 (14.3)	1964 (14.3)		92 (8.4)	184 (8.4)		742 (17.9)	1484 (17.9)	
70–74	1744 (25.4)	3488 (25.4)		237 (21.6)	474 (21.6)		830 (20.0)	1660 (20.0)	
75–79	1783 (26.0)	3566 (26.0)		314 (28.7)	628 (28.7)		560 (13.5)	1120 (13.5)	
80–84	931 (13.6)	1862 (13.6)		245 (22.4)	490 (22.4)		235 (5.7)	470 (5.7)	
85+	300 (4.4)	600 (4.4)		105 (9.6)	210 (9.6)		48 (1.2)	96 (1.2)	
Sex			<0.001			<0.001			<0.001
Male	1069 (15.6)	2138 (15.6)		291 (26.6)	582 (26.6)		241 (5.8)	482 (5.8)	
Female	5789 (84.4)	11,578 (84.4)		805 (73.5)	1610 (73.5)		3908 (94.2)	7816 (94.2)	
**Income**			<0.001			<0.001			<0.001
1 (lowest)	1171 (17.1)	2342 (17.1)		186 (17.0)	372 (17.0)		737 (17.8)	1474 (17.8)	
2	829 (12.1)	1658 (12.1)		134 (12.2)	268 (12.2)		491 (11.8)	982 (11.8)	
3	1031 (15.0)	2062 (15.0)		172 (15.7)	344 (15.7)		639 (15.4)	1278 (15.4)	
4	1378 (20.1)	2756 (20.1)		212 (19.3)	424 (19.3)		885 (21.3)	1770 (21.3)	
5 (highest)	2449 (35.7)	4898 (35.7)		392 (35.8)	784 (35.8)		1397 (33.7)	2794 (33.7)	
**Region of residence**			<0.001			<0.001			<0.001
Urban	2107 (30.7)	4214 (30.7)		356 (32.5)	712 (32.5)		1621 (39.1)	3242 (39.1)	
Rural	4751 (69.3)	9502 (69.3)		740 (67.5)	1480 (67.5)		2528 (60.9)	5056 (60.9)	
**Obesity ^2^**			0.08			0.22			0.11
Underweight	295 (4.3)	418 (3.1)		98 (8.9)	85 (3.9)		124 (3.0)	211 (2.5)	
Normal	2543 (37.1)	4815 (35.1)		422 (38.5)	820 (37.4)		1600 (38.6)	2913 (35.1)	
Overweight	1698 (24.8)	3552 (25.9)		250 (22.8)	580 (26.5)		1111 (26.8)	2157 (26.0)	
Obese I	2071 (30.2)	4394 (32.0)		292 (26.6)	633 (28.9)		1195 (28.8)	2682 (32.3)	
Obese II	251 (3.7)	537 (3.9)		34 (3.1)	74 (3.4)		119 (2.9)	335 (4.0)	
**Smoking status**			0.04			0.13			0.02
Nonsmoker	6128 (89.4)	12,320 (89.8)		892 (81.4)	1846 (84.2)		3925 (94.6)	7857 (94.7)	
Past smoker	397 (5.8)	843 (6.2)		108 (9.9)	226 (10.3)		106 (2.6)	228 (2.8)	
Current smoker	333 (4.9)	553 (4.0)		96 (8.8)	120 (5.5)		118 (2.8)	213 (2.6)	
**Alcohol consumption**			0.01			0.11			0.00
<1 time a week	5129 (74.8)	10,189 (74.3)		825 (75.3)	1541 (70.3)		3208 (77.3)	6411 (77.3)	
≥1 time a week	1729 (25.2)	3527 (25.7)		271 (24.7)	651 (29.7)		941 (22.7)	1887 (22.7)	
**CCI score**			0.19			0.51			0.12
0	3468 (50.6)	8243 (60.1)		356 (32.5)	1225 (55.9)		2615 (63.0)	5704 (68.7)	
1	1562 (22.8)	2567 (18.7)		253 (23.1)	430 (19.6)		767 (18.5)	1316 (15.9)	
2	835 (12.2)	1392 (10.2)		186 (17.0)	243 (11.1)		387 (9.3)	657 (7.9)	
≥ 3	993 (14.5)	1514 (11.0)		301 (27.5)	294 (13.4)		380 (9.2)	621 (7.5)	
**Osteoporosis**	5861 (85.5)	11,221 (81.8)	0.09	859 (78.4)	1738 (79.3)	0.02	3389 (81.7)	6811 (82.1)	0.01
**PA**			0.09			0.09			0.02
Low PA	4311 (62.9)	8036 (58.6)		710 (64.8)	1322 (60.3)		2330 (56.2)	4751 (57.3)	
High PA	2547 (37.1)	5680 (41.4)		386 (35.2)	870 (39.7)		1819 (43.8)	3547 (42.8)	
**SD**			0.02			0.05			0.01
Short SD (<6.1 h)	3514 (51.2)	6912 (50.4)		511 (46.6)	1073 (49.0)		2074 (50.0)	4123 (49.7)	
Long SD (≥6.1 h)	3344 (48.8)	6804 (49.6)		585 (53.4)	1119 (51.1)		2075 (50.0)	4175 (50.3)	
**PA + SD**			0.09			0.11			0.02
Low PA + Short SD	2316 (33.8)	4232 (30.9)		347 (31.7)	681 (31.1)		1196 (28.8)	2438 (29.4)	
Low PA + Long SD	1995 (29.1)	3804 (27.7)		363 (33.1)	641 (29.2)		1134 (27.3)	2313 (27.9)	
High PA + Short SD	1198 (17.5)	2680 (19.5)		164 (15.0)	392 (17.9)		878 (21.2)	1685 (20.3)	
High PA + Long SD	1349 (19.7)	3000 (21.9)		222 (20.3)	478 (21.8)		941 (22.7)	1862 (22.4)	

CCI, Charlson Comorbidity Index; Low PA, low-intensity physical activity; High PA, moderate- to high-intensity physical activity; PA, physical activity; SD, sunshine duration. ^1^ Absolute value of standardized difference. Differences in means or proportions divided by standard error; imbalance defined as absolute value > 0.20. ^2^ Obesity (BMI, body mass index, kg/m^2^) was categorized as < 18.5 (underweight), ≥ 18.5 to < 23 (normal), ≥ 23 to < 25 (overweight), ≥ 25 to < 30 (obese I), or ≥ 30 (obese II).

**Table 2:** Odds ratios (95% confidence intervals) of physical activity and sunshine duration for vertebral fracture with subgroup analyses according to age, sex, and osteoporosis history (reference: Low PA + Short SD).

Characteristics	Vertebral Fracture	Control I	OR (95% Confidence Intervals) for Vertebral Fracture				*p*-Value for Interaction *
	(Exposure/Total, %)	(Exposure/Total, %)	Crude ^1^	*p*-Value *	Adjusted ^1,2^	*p*-Value *	
**Total participants (*n* = 20,574)**							
Low PA + Long SD	1995/6858 (29.1)	3804/13,716 (27.7)	0.95 (0.88–1.03)	0.21	0.98 (0.90–1.05)	0.52	
High PA + Short SD	1198/6858 (17.5)	2680/13,716 (19.5)	0.81 (0.75–0.89)	<0.001 *	0.83 (0.76–0.91)	<0.001 *	
High PA + Long SD	1349/6858 (19.7)	3000/13,716 (21.9)	0.81 (0.75–0.89)	<0.001 *	0.84 (0.77–0.92)	<0.001 *	
**Age < 60 years old (*n* = 6300)**							0.95
Low PA + Long SD	561/2100 (26.7)	1145/4200 (27.3)	0.83 (0.72–0.96)	0.011 *	0.83 (0.72–0.97)	0.015 *	
High PA + Short SD	400/2100 (19.1)	886/4200 (21.1)	0.77 (0.66–0.90)	0.001 *	0.79 (0.68–0.92)	0.002 *	
High PA + Long SD	471/2100 (22.4)	1022/4200 (24.3)	0.78 (0.67–0.91)	0.001 *	0.79 (0.68–0.92)	0.002 *	
**Age ≥ 60 years old (*n* = 14,274)**							
Low PA + Long SD	1434/4758 (30.1)	2659/9516 (27.9)	1.01 (0.92–1.10)	0.89	1.04 (0.95–1.14)	0.39	
High PA + Short SD	798/4758 (16.8)	1794/9516 (18.9)	0.83 (0.75–0.92)	<0.001 *	0.85 (0.76–0.94)	0.002 *	
High PA + Long SD	878/4758 (18.5)	1978/9516 (20.8)	0.82 (0.74–0.91)	<0.001 *	0.86 (0.77–0.95)	0.004 *	
**Males (*n* = 3207)**							0.18
Low PA + Long SD	280/1069 (26.2)	501/2138 (23.4)	0.94 (0.77–1.16)	0.57	0.98 (0.80–1.21)	0.86	
High PA + Short SD	211/1069 (19.7)	503/2138 (23.5)	0.71 (0.58–0.88)	0.002 *	0.76 (0.62–0.95)	0.013 *	
High PA + Long SD	223/1069 (20.9)	528/2138 (24.7)	0.71 (0.57–0.88)	0.002 *	0.76 (0.61–0.95)	0.014 *	
**Females (*n* = 17,367)**							
Low PA + Long SD	1715/5789 (29.6)	3303/11,578 (28.5)	0.96 (0.88–1.04)	0.27	0.98 (0.90–1.06)	0.55	
High PA + Short SD	987/5789 (17.1)	2177/11,578 (18.8)	0.84 (0.76–0.92)	<0.001 *	0.85 (0.77–0.93)	0.001 *	
High PA + Long SD	1126/5789 (19.5)	2472/11,578 (21.4)	0.84 (0.76–0.92)	<0.001 *	0.86 (0.78–0.94)	0.001 *	
**Nonosteoporosis (*n* = 3492)**							0.008 *
Low PA + Long SD	294/997 (29.5)	695/2495 (27.9)	0.89 (0.73–1.07)	0.21	0.88 (0.72–1.07)	0.19	
High PA + Short SD	183/997 (18.4)	475/2495 (19.0)	0.81 (0.65–1.00)	0.05	0.79 (0.63–0.98)	0.03	
High PA + Long SD	189/997 (19.0)	632/2495 (25.3)	0.63 (0.51–0.77)	<0.001 *	0.60 (0.48–0.74)	<0.001 *	
**Osteoporosis (*n* = 17,082)**							
Low PA + Long SD	1701/5861 (29.0)	3109/11,221 (27.7)	0.98 (0.90–1.06)	0.55	0.99 (0.91–1.08)	0.87	
High PA + Short SD	1015/5861 (17.3)	2205/11,221 (19.7)	0.82 (0.75–0.90)	<0.001 *	0.84 (0.76–0.92)	<0.001 *	
High PA + Long SD	1160/5861 (19.8)	2368/11,221 (21.1)	0.87 (0.80–0.96)	0.003 *	0.90 (0.82–0.98)	0.02	

CCI, Charlson Comorbidity Index; Low PA, low-intensity physical activity; High PA, moderate- to high-intensity physical activity; OR, odds ratio; PA, physical activity; SD, sunshine duration. * Conditional logistic regression for total participants and subgroup analyses according to age and sex; unconditional logistic regression for subgroup analyses according to osteoporosis history; significance at < 0.05 with Bonferroni correction (α = 0.05/3). ^1^ Models were stratified by age, sex, income, and region of residence except for subgroup analyses according to osteoporosis history. ^2^ For conditional logistic regression, the adjusted model was adjusted for obesity, smoking, alcohol consumption, osteoporosis, and CCI scores, and for the unconditional logistic regression, the adjusted model was adjusted for the above variables plus age, sex, income, and region of residence.

**Table 3:** Odds ratios (95% confidence intervals) of physical activity and sunshine duration for hip fracture with subgroup analyses according to age, sex, and osteoporosis history (reference: Low PA + Short SD).

Characteristics	Hip Fracture	Control II	OR (95% Confidence Intervals) for Hip Fracture				*p*-Value for Interaction *
	(Exposure/Total, %)	(Exposure/Total, %)	Crude ^1^	*p*-Value *	Adjusted ^1,2^	*p*-Value *	
**Total participants (*n* = 3288)**							
Low PA + Long SD	363/1096 (33.1)	641/2192 (29.2)	1.12 (0.93–1.35)	0.24	1.17 (0.96–1.42)	0.12	
High PA + Short SD	164/1096 (15.0)	392/2192 (17.9)	0.82 (0.66–1.03)	0.08	0.83 (0.66–1.04)	0.11	
High PA + Long SD	222/1096 (20.3)	478/2192 (21.8)	0.92 (0.74–1.14)	0.44	0.97 (0.78–1.21)	0.80	
**Age < 60 years old (*n* = 585)**							0.54
Low PA + Long SD	63/195 (32.3)	99/390 (25.4)	1.32 (0.80–2.17)	0.28	1.30 (0.76–2.22)	0.34	
High PA + Short SD	35/195 (18.0)	87/390 (22.3)	0.82 (0.49–1.37)	0.44	0.81 (0.47–1.39)	0.44	
High PA + Long SD	46/195 (23.6)	102/390 (26.2)	0.94 (0.56–1.59)	0.82	0.81 (0.46–1.45)	0.49	
**Age ≥ 60 years old (*n* = 2703)**							
Low PA + Long SD	300/901 (33.3)	542/1802 (30.1)	1.09 (0.89–1.34)	0.42	1.15 (0.93–1.43)	0.19	
High PA + Short SD	129/901 (14.3)	305/1802 (16.9)	0.83 (0.64–1.06)	0.13	0.83 (0.64–1.08)	0.16	
High PA + Long SD	176/901 (19.5)	376/1802 (20.9)	0.92 (0.73–1.17)	0.49	0.99 (0.83–1.27)	0.95	
**Males (*n* = 873)**							0.35
Low PA + Long SD	102/291 (35.1)	149/582 (25.6)	1.50 (1.01–2.22)	0.04	1.45 (0.95–2.19)	0.08	
High PA + Short SD	50/291 (17.2)	125/582 (21.5)	0.85 (0.56–1.31)	0.47	0.81 (0.52–1.26)	0.35	
High PA + Long SD	65/291 (22.3)	151/582 (26.0)	0.96 (0.63–1.47)	0.85	0.90 (0.57–1.40)	0.64	
**Females (*n* = 2415)**							
Low PA + Long SD	261/805 (32.4)	492/1610 (30.6)	1.02 (0.82–1.27)	0.86	1.10 (0.88–1.38)	0.41	
High PA + Short SD	114/805 (14.2)	267/1610 (16.6)	0.82 (0.63–1.07)	0.14	0.85 (0.64–1.11)	0.22	
High PA + Long SD	157/805 (19.5)	327/1610 (20.3)	0.92 (0.72–1.18)	0.52	1.02 (0.78–1.32)	0.91	
**Nonosteoporosis (*n* = 691)**							0.11
Low PA + Long SD	88/237 (37.1)	137/454 (30.2)	1.15 (0.77–1.71)	0.50	1.34 (0.86–2.08)	0.19	
High PA + Short SD	35/237 (14.8)	88/454 (19.4)	0.71 (0.44–1.16)	0.17	0.74 (0.44–1.24)	0.25	
High PA + Long SD	44/237 (18.6)	104/454 (22.9)	0.76 (0.48–1.19)	0.23	0.76 (0.46–1.28)	0.30	
**Osteoporosis (*n* = 2597)**							
Low PA + Long SD	275/859 (32.0)	504/1738 (29.0)	1.10 (0.89–1.35)	0.39	1.13 (0.91–1.40)	0.26	
High PA + Short SD	129/859 (15.0)	304/1738 (17.5)	0.85 (0.66–1.10)	0.21	0.85 (0.66–1.10)	0.22	
High PA + Long SD	178/859 (20.7)	374/1738 (21.5)	0.96 (0.76–1.20)	0.70	1.02 (0.80–1.30)	0.90	

CCI, Charlson Comorbidity Index; Low PA, low-intensity physical activity; High PA, moderate- to high-intensity physical activity; OR, odds ratio; PA, physical activity; SD, sunshine duration. * Conditional logistic regression for total participants and subgroup analyses according to age and sex; unconditional logistic regression for subgroup analyses according to osteoporosis history, significance at < 0.05 with Bonferroni correction (α = 0.05/3). ^1^ Models were stratified by age, sex, income, and region of residence except for subgroup analyses according to osteoporosis history. ^2^ For conditional logistic regression, the adjusted model was adjusted for obesity, smoking, alcohol consumption, osteoporosis, and CCI scores, and for the unconditional logistic regression, the adjusted model was adjusted for the above variables plus age, sex, income, and region of residence.

**Table 4:** Odds ratios (95% confidence intervals) of physical activity and sunshine duration for distal radius fracture with subgroup analyses according to age, sex, and osteoporosis history (reference: Low PA + Short SD).

Characteristics	Distal Radius Fracture	Control III	OR (95% Confidence Intervals) for Distal Radius Fracture				*p*-Value for Interaction *
	(Exposure/Total, %)	(Exposure/Total, %)	Crude ^1^	*p*-Value *	Adjusted ^1,2^	*p*-Value *	
**Total participants (*n* = 12,447)**							
Low PA + Long SD	1134/4149 (27.3)	2313/8298 (27.9)	1.00 (0.90–1.11)	0.99	1.00 (0.90–1.11)	0.95	
High PA + Short SD	878/4149 (21.2)	1685/8298 (20.3)	1.06 (0.95–1.18)	0.27	1.07 (0.96–1.19)	0.25	
High PA + Long SD	941/4149 (23.0)	1862/8298 (22.4)	1.03 (0.92–1.15)	0.59	1.03 (0.92–1.15)	0.63	
**Age < 60 years old (*n* = 7428)**							0.736
Low PA + Long SD	672/2476 (27.1)	1337/4952 (27.0)	1.04 (0.91–1.19)	0.55	1.03 (0.90–1.18)	0.66	
High PA + Short SD	547/2476 (22.1)	1090/4952 (22.0)	1.04 (0.90–1.19)	0.61	1.04 (0.90–1.19)	0.63	
High PA + Long SD	589/2476 (23.8)	1146/4952 (23.1)	1.07 (0.93–1.23)	0.37	1.05 (0.91–1.21)	0.49	
**Age ≥ 60 years old (*n* = 5019)**							
Low PA + Long SD	462/1673 (27.6)	976/3346 (29.2)	0.94 (0.81–1.10)	0.46	0.95 (0.81–1.12)	0.53	
High PA + Short SD	331/1673 (19.8)	595/3346 (17.8)	1.12 (0.94–1.32)	0.21	1.13 (0.95–1.34)	0.17	
High PA + Long SD	352/1673 (21.0)	716/3346 (21.4)	0.98 (0.82–1.16)	0.79	0.99 (0.83–1.18)	0.92	
**Males (*n* = 723)**							0.062
Low PA + Long SD	71/241 (29.5)	130/482 (27.0)	0.93 (0.69–1.44)	0.74	0.92 (0.59–1.43)	0.71	
High PA + Short SD	47/241 (19.5)	99/482 (20.5)	0.83 (0.53–1.31)	0.43	0.85 (0.53–1.34)	0.47	
High PA + Long SD	54/241 (22.4)	132/482 (27.4)	0.69 (0.44–1.11)	0.13	0.70 (0.44–1.12)	0.14	
**Females (*n* = 11,724)**							
Low PA + Long SD	1063/3908 (27.2)	2183/7816 (27.9)	1.00 (0.90–1.11)	0.97	1.00 (0.90–1.11)	0.95	
High PA + Short SD	831/3908 (21.3)	1586/7816 (20.3)	1.08 (0.97–1.20)	0.18	1.08 (0.97–1.21)	0.17	
High PA + Long SD	887/3908 (22.7)	1730/7816 (22.1)	1.06 (0.94–1.18)	0.34	1.05 (0.94–1.18)	0.42	
**Nonosteoporosis (*n* = 2247)**							0.016 *
Low PA + Long SD	216/760 (28.4)	433/1487 (29.1)	0.87 (0.69–1.10)	0.24	0.87 (0.69–1.11)	0.26	
High PA + Short SD	153/760 (20.1)	287/1487 (19.3)	0.93 (0.72–1.20)	0.58	0.93 (0.72–1.21)	0.60	
High PA + Long SD	166/760 (21.8)	374/1487 (25.2)	0.78 (0.61–0.99)	0.04	0.78 (0.61–1.01)	0.06	
**Osteoporosis (*n* = 10,200)**							
Low PA + Long SD	918/3389 (27.1)	1880/6811 (27.6)	1.03 (0.92–1.15)	0.62	0.87 (0.69–1.11)	0.26	
High PA + Short SD	725/3389 (21.4)	1398/6811 (20.5)	1.09 (0.97–1.23)	0.14	0.93 (0.72–1.21)	0.60	
High PA + Long SD	775/3389 (22.9)	1488/6811 (21.9)	1.10 (0.98–1.23)	0.12	0.78 (0.61–1.01)	0.06	

CCI, Charlson Comorbidity index; Low PA, low-intensity physical activity; High PA, moderate- to high-intensity physical activity; OR, odds ratio; PA, physical activity; SD, sunshine duration. * Conditional logistic regression for total participants and subgroup analyses according to age and sex; unconditional logistic regression for subgroup analyses according to osteoporosis history, significance at < 0.05 with Bonferroni correction (α = 0.05/3). ^1^ Models were stratified by age, sex, income, and region of residence except for subgroup analyses according to osteoporosis history. ^2^ For conditional logistic regression, the adjusted model was adjusted for obesity, smoking, alcohol consumption, osteoporosis, and CCI scores, and for the unconditional logistic regression, the adjusted model was adjusted for the above variables plus age, sex, income, and region of residence.

## Data Availability

Restrictions apply to the availability of these data. Data were obtained from the Korean National Health Insurance Sharing Service (NHISS) and are available at https://nhiss.nhis.or.kr (accessed on 19 January 2022) with the permission of the NHIS.

## Supplementary Materials

The following are available online at https://www.mdpi.com/article/10.3390/jpm12020164/s1: Table S1: Subgroup analyses regarding odds ratios (95% confidence intervals) of physical activity and sunshine duration for vertebral fracture according to income, region of residence, obesity, smoking status, and alcohol consumption (reference: Low PA + Short SD), Table S2: Subgroup analyses regarding odds ratios (95% confidence intervals) of physical activity and sunshine duration for hip fracture according to income, region of residence, obesity, smoking status, and alcohol consumption (reference: Low PA + Short SD), Table S3: Subgroup analyses regarding odds ratios (95% confidence intervals) of physical activity and sunshine duration for distal radius fracture according to income, region of residence, obesity, smoking status, and alcohol consumption (reference: Low PA + Short SD), Table S4: Odds ratios (95% confidence intervals) of moderate- to high-intensity physical activity for vertebral fracture and of long sunshine duration for vertebral fracture with subgroup analyses according to age, sex, income, region of residence, sunshine duration (or physical activity) status of obesity, smoking, and alcohol consumption, and osteoporosis history, Table S5: Odds ratios (95% confidence intervals) of moderate- to high-intensity physical activity for vertebral fracture and of long sunshine duration for hip fracture with subgroup analyses according to age, sex, income, region of residence, sunshine duration (or physical activity) status of obesity, smoking, and alcohol consumption, and osteoporosis history, Table S6: Odds ratios (95% confidence intervals) of moderate- to high-intensity physical activity for distal radius fracture and of long sunshine duration for distal radius fracture with subgroup analyses according to age, sex, income, region of residence, sunshine duration (or physical activity) status of obesity, smoking, and alcohol consumption, and osteoporosis history.
