# Supplementary material for: Physical Activity, Sunshine Duration, and Osteoporotic Fractures: A Nested Case-Control Study
Source: J Pers Med. 2022 Jan 26;12(2):164. doi: 10.3390/jpm12020164 (PMC8878966; doi:10.3390/jpm12020164)
Supplement: Supplementary file 1 [file jpm-12-00164-s001.zip › jpm-1516121-supplementary.pdf]

Table S1. Subgroup analyses regarding odds ratios (95% confidence intervals) of physical activity and sunshine duration for vertebral fracture according to income, region of residence, obesity, smoking status, and alcohol consumption (reference: Low PA + Short SD).

Characteristics	Vertebral Fracture	Control I	OR (95% Confidence Intervals) for Vertebral Fracture				*P* value
	(exposure/total, %)	(exposure/total, %)	Crude†	*P* value	Adjusted†‡	*P* value	For Interaction
Low income (*n* = 9093)							
Low PA + Long SD	883/3031 (29.1)	1683/6062 (27.8)	0.93 (0.83–1.04)	0.220	0.95 (0.84–1.06)	0.352	
High PA + Short SD	506/3031 (16.7)	1187/6062 (19.6)	0.76 (0.67–0.86)	<0.001*	0.77 (0.68–0.87)	<0.001*	
High PA + Long SD	544/3031 (18.0)	1229/6062 (20.3)	0.78 (0.69–0.89)	<0.001*	0.80 (0.70–0.91)	0.001*	
High income (*n* = 11,481)							0.311
Low PA + Long SD	1112/3827 (29.1)	2121/7654 (27.7)	0.97 (0.88–1.08)	0.585	1.00 (0.90–1.11)	0.994	
High PA + Short SD	692/3827 (18.1)	1493/7654 (19.5)	0.86 (0.77–0.97)	0.010*	0.89 (0.79–0.99)	0.038	
High PA + Long SD	805/3827 (21.0)	1771/7654 (23.1)	0.84 (0.75–0.94)	0.002*	0.88 (0.78–0.98)	0.024	
Urban (*n* = 6321)							
Low PA + Long SD	802/2107 (38.1)	1518/4214 (36.0)	0.93 (0.79–1.08)	0.333	0.93 (0.79–1.09)	0.346	
High PA + Short SD	246/2107 (11.7)	579/4214 (13.7)	0.74 (0.61–0.90)	0.003*	0.76 (0.62–0.92)	0.005*	
High PA + Long SD	685/2107 (32.5)	1462/4214 (34.7)	0.82 (0.70–0.96)	0.013*	0.84 (0.71–0.98)	0.030	
Rural (*n* = 14,253)							0.827
Low PA + Long SD	1193/4751 (25.1)	2286/9502 (24.1)	0.96 (0.88–1.05)	0.391	0.99 (0.91–1.09)	0.875	
High PA + Short SD	952/4751 (20.0)	2101/9502 (22.1)	0.83 (0.76–0.92)	<0.001*	0.85 (0.78–0.94)	0.001*	
High PA + Long SD	664/4751 (14.0)	1538/9502 (16.2)	0.79 (0.71–0.88)	<0.001*	0.82 (0.74–0.91)	<0.001*	
Underweight (*n* = 713)							
Low PA + Long SD	92/295 (31.2)	124/418 (29.7)	0.99 (0.69–1.43)	0.954	1.09 (0.74–1.61)	0.678	
High PA + Short SD	50/295 (17.0)	72/418 (17.2)	0.93 (0.60–1.44)	0.731	0.96 (0.62–1.51)	0.870	
High PA + Long SD	45/295 (15.3)	78/418 (18.7)	0.77 (0.49–1.20)	0.247	0.81 (0.51–1.30)	0.381	
Normal weight (*n* = 7358)							
Low PA + Long SD	724/2543 (28.5)	1316/4815 (27.3)	0.92 (0.82–1.04)	0.194	0.95 (0.84–1.08)	0.453	
High PA + Short SD	443/2543 (17.4)	904/4815 (18.8)	0.82 (0.71–0.95)	0.006*	0.84 (0.73–0.97)	0.016*	
High PA + Long SD	492/2543 (19.4)	1114/4815 (23.1)	0.74 (0.65–0.85)	<0.001*	0.78 (0.68–0.90)	0.001*	
Overweight (*n* = 5250)							0.084
Low PA + Long SD	492/1698 (29.0)	976/3552 (27.5)	0.95 (0.82–1.11)	0.534	0.97 (0.83–1.13)	0.684	
High PA + Short SD	297/1698 (17.5)	724/3552 (20.4)	0.78 (0.66–0.92)	0.003*	0.80 (0.67–0.95)	0.009*	
High PA + Long SD	354/1698 (20.9)	802/3552 (22.6)	0.84 (0.71–0.98)	0.029	0.86 (0.72–1.02)	0.076	
Obese (*n* = 7253)							
Low PA + Long SD	687/2322 (29.6)	1388/4931 (28.2)	1.00 (0.88–1.14)	0.973	1.01 (0.89–1.15)	0.884	
High PA + Short SD	408/2322 (17.6)	980/4931 (19.9)	0.84 (0.73–0.97)	0.020	0.84 (0.73–0.98)	0.022	
High PA + Long SD	458/2322 (19.7)	1006/4931 (20.4)	0.92 (0.80–1.06)	0.255	0.91 (0.78–1.05)	0.198	
Nonsmoker (*n* = 18,448)							
Low PA + Long SD	1792/6128 (29.2)	3435/12320 (27.9)	0.98 (0.90–1.05)	0.518	0.99 (0.91–1.07)	0.818	
High PA + Short SD	1066/6128 (17.4)	2353/12320 (19.1)	0.85 (0.77–0.93)	<0.001*	0.86 (0.79–0.94)	0.001*	
High PA + Long SD	1202/6128 (19.6)	2669/12320 (21.7)	0.84 (0.77–0.92)	<0.001*	0.86 (0.78–0.94)	0.001*	
Past or current smoker (*n* = 2126)							0.192
Low PA + Long SD	203/730 (27.8)	369/1396 (26.4)	0.82 (0.65–1.04)	0.095	0.84 (0.66–1.08)	0.166	
High PA + Short SD	132/730 (18.1)	327/1396 (23.4)	0.60 (0.46–0.78)	<0.001*	0.61 (0.47–0.79)	<0.001*	
High PA + Long SD	147/730 (20.1)	331/1396 (23.7)	0.66 (0.51–0.85)	0.001*	0.70 (0.53–0.91)	0.008*	
< 1 time a week alcohol consumption (*n* = 15,318)							
Low PA + Long SD	1394/5129 (27.2)	2617/10189 (25.7)	0.97 (0.89-1.06)	0.500	1.00 (0.91–1.09)	0.945	
High PA + Short SD	942/5129 (18.4)	2133/10189 (20.9)	0.81 (0.73-0.89)	<0.001*	0.82 (0.75–0.90)	<0.001*	
High PA + Long SD	904/5129 (17.6)	1996/10189 (19.6)	0.83 (0.75-0.91)	<0.001*	0.85 (0.77–0.94)	0.002*	
≥ 1 time a week alcohol consumption (*n* = 5256)							0.782
Low PA + Long SD	601/1729 (34.8)	1187/3527 (33.7)	0.94 (0.80–1.09)	0.394	0.91 (0.78–1.07)	0.266	
High PA + Short SD	256/1729 (14.8)	547/3527 (15.5)	0.87 (0.72–1.05)	0.133	0.87 (0.72–1.06)	0.167	
High PA + Long SD	445/1729 (25.7)	1004/3527 (28.5)	0.82 (0.70–0.96)	0.016*	0.80 (0.67–0.95)	0.010*	

Abbreviations: CCI, Charlson comorbidity index; Low PA, low-intensity physical activity; High PA, moderate- to high-intensity physical activity; OR, odds ratio; PA, physical activity; SD, sunshine duration * Conditional logistic regression for subgroup analyses according to income, and region of residence, un-conditional logistic regression for subgroup analyses according to obesity, smoking, and alcohol consumption, significance at < 0.05 with Bonferroni correction (α = 0.05/3). † In subgroup analyses according to income and region of residence, models were stratified by age, sex, income, and region of residence. In subgroup analyses according to obesity, smoking, and alcohol consumption were not stratified. ‡ For conditional logistic regression, the adjusted model was adjusted for obesity, smoking, alcohol consumption, osteoporosis, and CCI scores, and for the un-conditional logistic regression, the adjusted model was adjusted for above variables plus age, sex, income, and region of residence.

Table S2. Subgroup analyses regarding odds ratios (95% confidence intervals) of physical activity and sunshine duration for hip fracture according to income, region of residence, obesity, smoking status, and alcohol consumption (reference: Low PA + Short SD).

Characteristics	Hip fracture	Control II	OR (95% Confidence Intervals) for Hip Fracture				*P* value
	(exposure/total, %)	(exposure/total, %)	Crude†	*P* value	Adjusted†‡	*P* value	for Interaction
Low income (*n* = 1476)							0.976
Low PA + Long SD	155/492 (31.5)	279/984 (28.4)	1.13 (0.85–1.50)	0.396	1.19 (0.88–1.60)	0.255	
High PA + Short SD	74/492 (15.0)	164/984 (16.7)	0.91 (0.65–1.27)	0.578	0.93 (0.66–1.31)	0.672	
High PA + Long SD	97/492 (19.7)	206/984 (20.9)	0.96 (0.69–1.33)	0.800	1.00 (0.71–1.41)	0.996	
High income (*n* = 1812)							
Low PA + Long SD	208/604 (34.4)	362/1208 (30.0)	1.11 (0.86–1.43)	0.438	1.16 (0.89–1.51)	0.277	
High PA + Short SD	90/604 (14.9)	228/1208 (18.9)	0.75 (0.56–1.02)	0.068	0.76 (0.56–1.04)	0.085	
High PA + Long SD	125/604 (20.7)	272/1208 (22.5)	0.89 (0.66–1.18)	0.404	0.93 (0.69–1.25)	0.632	
Urban (*n* = 1068)							0.424
Low PA + Long SD	141/356 (39.6)	254/712 (35.7)	1.00 (0.69–1.44)	0.979	1.08 (0.73–1.58)	0.704	
High PA + Short SD	44/356 (12.4)	98/712 (13.8)	0.81 (0.51–1.28)	0.363	0.83 (0.52–1.34)	0.451	
High PA + Long SD	103/356 (28.9)	238/712 (33.4)	0.78 (0.53–1.14)	0.200	0.85 (0.57–1.27)	0.423	
Rural (*n* = 2220)							
Low PA + Long SD	222/740 (30.0)	387/1480 (26.2)	1.16 (0.93–1.45)	0.199	1.21 (0.96–1.52)	0.115	
High PA + Short SD	120/740 (16.2)	294/1480 (19.9)	0.82 (0.63–1.06)	0.122	0.83 (0.64–1.08)	0.166	
High PA + Long SD	119/740 (16.1)	240/1480 (16.2)	1.00 (0.77–1.30)	0.993	1.05 (0.80–1.39)	0.724	
Underweight (*n* = 183)							0.825
Low PA + Long SD	26/98 (26.5)	30/85 (35.3)	0.69 (0.33–1.43)	0.315	0.75 (0.33–1.73)	0.500	
High PA + Short SD	11/98 (11.2)	16/85 (18.8)	0.55 (0.22–1.37)	0.197	0.56 (0.20–1.56)	0.266	
High PA + Long SD	27/98 (27.6)	12/85 (14.1)	1.79 (0.77–4.17)	0.180	1.86 (0.67–5.12)	0.231	
Normal weight (*n* = 1242)							
Low PA + Long SD	145/422 (34.4)	233/820 (28.4)	1.16 (0.86–1.55)	0.337	1.19 (0.87–1.63)	0.268	
High PA + Short SD	61/422 (14.5)	158/820 (19.3)	0.72 (0.50–1.03)	0.072	0.72 (0.49–1.04)	0.082	
High PA + Long SD	83/422 (19.7)	182/820 (22.2)	0.85 (0.61–1.18)	0.330	0.86 (0.61–1.23)	0.415	
Overweight (*n* = 830)							
Low PA + Long SD	85/250 (34.0)	159/580 (27.4)	1.22 (0.84–1.76)	0.303	1.29 (0.88–1.91)	0.197	
High PA + Short SD	40/250 (16.0)	102/580 (17.6)	0.89 (0.57–1.40)	0.620	0.94 (0.59–1.50)	0.791	
High PA + Long SD	45/250 (18.0)	137/580 (23.6)	0.75 (0.49–1.15)	0.182	0.83 (0.53–1.30)	0.414	
Obese (*n* = 1033)							
Low PA + Long SD	107/326 (32.8)	219/707 (31.0)	1.10 (0.79–1.53)	0.574	1.15 (0.81–1.62)	0.439	
High PA + Short SD	52/326 (16.0)	116/707 (16.4)	1.01 (0.67–1.51)	0.967	1.01 (0.67–1.53)	0.968	
High PA + Long SD	67/326 (20.6)	147/707 (20.8)	1.03 (0.71–1.49)	0.895	1.10 (0.74–1.65)	0.632	
Nonsmoker (*n* = 2738)							0.837
Low PA + Long SD	296/892 (33.2)	561/1846 (30.4)	1.06 (0.87–1.30)	0.551	1.12 (0.91–1.38)	0.293	
High PA + Short SD	132/892 (14.8)	317/1846 (17.2)	0.84 (0.66–1.07)	0.163	0.86 (0.66–1.11)	0.240	
High PA + Long SD	177/892 (19.8)	390/1846 (21.1)	0.91 (0.73–1.15)	0.438	0.96 (0.75–1.22)	0.737	
Past or current smoker (*n* = 550)							
Low PA + Long SD	67/204 (32.8)	80/346 (23.1)	1.44 (0.91–2.27)	0.118	1.58 (0.96–2.61)	0.071	
High PA + Short SD	32/204 (15.7)	75/346 (21.7)	0.73 (0.44–1.24)	0.243	0.77 (0.44–1.34)	0.349	
High PA + Long SD	45/204 (22.1)	88/346 (25.4)	0.88 (0.54–1.42)	0.595	1.04 (0.61–1.77)	0.882	
< 1 time a week alcohol consumption (*n* = 2366)							0.216
Low PA + Long SD	265/825 (32.1)	420/1541 (27.3)	1.15 (0.93–1.41)	0.210	1.18 (0.94–1.47)	0.150	
High PA + Short SD	122/825 (14.8)	292/1541 (19.0)	0.76 (0.59–0.98)	0.034	0.76 (0.58–0.99)	0.038*	
High PA + Long SD	153/825 (18.6)	312/1541 (20.3)	0.89 (0.70–1.13)	0.342	0.91 (0.70–1.17)	0.462	
≥ 1 time a week alcohol consumption (*n* = 922)							
Low PA + Long SD	98/271 (36.2)	221/651 (34.0)	1.17 (0.81–1.71)	0.407	1.17 (0.79–1.73)	0.442	
High PA + Short SD	42/271 (15.5)	100/651 (15.4)	1.11 (0.70–1.77)	0.657	1.12 (0.69–1.81)	0.647	
High PA + Long SD	69/271 (25.5)	166/651 (25.5)	1.10 (0.73–1.65)	0.646	1.14 (0.74–1.76)	0.558	

Abbreviations: CCI, Charlson comorbidity index; Low PA, low-intensity physical activity; High PA, moderate- to high-intensity physical activity; OR, odds ratio; PA, physical activity; SD, sunshine duration * Conditional logistic regression for subgroup analyses according to income, and region of residence, un-conditional logistic regression for subgroup analyses according to obesity, smoking, and alcohol consumption, significance at < 0.05 with Bonferroni correction ($\alpha = 0.05/3$). † In subgroup analyses according to income and region of residence, models were stratified by age, sex, income, and region of residence. In subgroup analyses according to obesity, smoking, and alcohol consumption were not stratified. ‡ For conditional logistic regression, the adjusted model was adjusted for obesity, smoking, alcohol consumption, osteoporosis, and CCI scores, and for the un-conditional logistic regression, the adjusted model was adjusted for above variables plus age, sex, income, and region of residence.

Table S3. Subgroup analyses regarding odds ratios (95% confidence intervals) of physical activity and sunshine duration for distal radius fracture according to income, region of residence, obesity, smoking status, and alcohol consumption (reference: Low PA + Short SD).

Characteristics	Distal radius fracture (exposure/total, %)	Control III (exposure/total, %)	OR (95% confidence intervals) for Distal Radius Fracture				*P* value for Interaction
			Crude†	*P* value	Adjusted†‡	*P* value	
Low income (*n* = 5601)							0.706
Low PA + Long SD	501/1867 (26.8)	1012/3734 (27.1)	0.99 (0.85–1.15)	0.866	1.00 (0.86–1.16)	0.975	
High PA + Short SD	411/1867 (22.0)	768/3734 (20.6)	1.07 (0.92–1.26)	0.380	1.08 (0.92–1.26)	0.345	
High PA + Long SD	381/1867 (20.4)	803/3734 (21.5)	0.95 (0.80–1.11)	0.500	0.96 (0.81–1.13)	0.615	
High income (*n* = 6846)							
Low PA + Long SD	633/2282 (27.7)	1301/4564 (28.5)	1.01 (0.88–1.16)	0.872	1.00 (0.87–1.15)	0.995	
High PA + Short SD	467/2282 (20.5)	917/4564 (20.1)	1.06 (0.91–1.22)	0.472	1.05 (0.91–1.22)	0.506	
High PA + Long SD	560/2282 (24.5)	1059/4564 (23.2)	1.10 (0.95–1.28)	0.198	1.09 (0.94–1.26)	0.267	
Urban (*n* = 4863)							0.149
Low PA + Long SD	559/1621 (34.5)	1172/3242 (36.2)	1.03 (0.86–1.23)	0.764	1.02 (0.85–1.23)	0.831	
High PA + Short SD	262/1621 (16.2)	471/3242 (14.5)	1.20 (0.97–1.49)	0.094	1.20 (0.97–1.49)	0.091	
High PA + Long SD	551/1621 (34.0)	1062/3242 (32.8)	1.12 (0.93–1.35)	0.231	1.11 (0.92–1.33)	0.282	
Rural (*n* = 7584)							
Low PA + Long SD	575/2528 (22.8)	1141/5056 (22.6)	1.01 (0.89–1.15)	0.861	1.01 (0.89–1.15)	0.833	
High PA + Short SD	616/2528 (24.4)	1214/5056 (24.0)	1.02 (0.90–1.15)	0.772	1.02 (0.90–1.16)	0.757	
High PA + Long SD	390/2528 (15.4)	800/5056 (15.8)	0.98 (0.85–1.13)	0.769	0.99 (0.85–1.14)	0.864	
Underweight (*n* = 335)							0.806
Low PA + Long SD	34/124 (27.4)	73/211 (34.6)	0.60 (0.34-1.04)	0.068	0.63 (0.35–1.14)	0.126	
High PA + Short SD	22/124 (17.7)	32/211 (15.2)	0.88 (0.45-1.70)	0.700	0.78 (0.39–1.56)	0.488	
High PA + Long SD	21/124 (16.9)	46/211 (21.8)	0.58 (0.31-1.11)	0.099	0.59 (0.29–1.22)	0.155	
Normal weight (*n* = 4513)							
Low PA + Long SD	461/1600 (28.8)	803/2913 (27.6)	1.16 (0.99–1.37)	0.070	1.18 (1.00–1.40)	0.052	
High PA + Short SD	339/1600 (21.2)	586/2913 (20.1)	1.17 (0.98–1.40)	0.079	1.18 (0.99–1.42)	0.063	
High PA + Long SD	377/1600 (23.6)	667/2913 (22.9)	1.15 (0.96–1.36)	0.122	1.17 (0.98–1.40)	0.081	
Overweight (*n* = 3268)							
Low PA + Long SD	293/1111 (26.4)	576/2157 (26.7)	0.97 (0.80–1.18)	0.742	0.92 (0.75–1.12)	0.402	
High PA + Short SD	226/1111 (20.3)	463/2157 (21.5)	0.93 (0.76–1.14)	0.486	0.93 (0.76–1.15)	0.514	
High PA + Long SD	262/1111 (23.6)	490/2157 (22.7)	1.02 (0.83–1.24)	0.865	0.96 (0.78–1.19)	0.723	
Obese (*n* = 4331)							
Low PA + Long SD	346/1314 (26.3)	861/3017 (28.5)	0.91 (0.76–1.08)	0.262	0.93 (0.78–1.11)	0.436	
High PA + Short SD	291/1314 (22.2)	604/3017 (20.0)	1.09 (0.91–1.31)	0.375	1.09 (0.91–1.31)	0.353	
High PA + Long SD	281/1314 (21.4)	659/3017 (21.8)	0.96 (0.80–1.16)	0.675	0.99 (0.82–1.20)	0.932	
Nonsmoker (*n* = 11,782)							0.485
Low PA + Long SD	1060/3925 (27.0)	2191/7857 (27.9)	0.99 (0.89–1.10)	0.835	0.99 (0.89–1.10)	0.867	
High PA + Short SD	834/3925 (21.3)	1586/7857 (20.2)	1.08 (0.96–1.20)	0.195	1.08 (0.97–1.21)	0.167	
High PA + Long SD	891/3925 (22.7)	1749/7857 (22.3)	1.04 (0.94–1.16)	0.456	1.05 (0.93–1.17)	0.439	
Past or current smoker (*n* = 665)							
Low PA + Long SD	74/224 (33.0)	122/441 (27.7)	1.16 (0.75–1.79)	0.505	1.08 (0.69–1.70)	0.748	
High PA + Short SD	44/224 (19.6)	99/441 (22.5)	0.85 (0.53–1.37)	0.505	0.83 (0.51–1.36)	0.464	
High PA + Long SD	50/224 (22.3)	113/441 (25.6)	0.85 (0.53–1.35)	0.478	0.79 (0.48–1.29)	0.346	
< 1 time a week alcohol consumption (*n* = 9619)							0.895
Low PA + Long SD	783/3,208 (24.4)	1,676/6,411 (26.1)	0.93 (0.83–1.04)	0.205	0.93 (0.83–1.05)	0.223	
High PA + Short SD	726/3,208 (22.6)	1,392/6,411 (21.7)	1.04 (0.92–1.17)	0.537	1.05 (0.93–1.18)	0.468	
High PA + Long SD	672/3,208 (21.0)	1,300/6,411 (20.3)	1.03 (0.91–1.16)	0.647	1.04 (0.91–1.17)	0.590	
≥ 1 time a week alcohol consumption (*n* = 2828)							
Low PA + Long SD	351/941 (37.3)	637/1887 (33.8)	1.29 (1.03–1.61)	0.026	1.28 (1.01–1.61)	0.039	
High PA + Short SD	152/941 (16.2)	293/1887 (15.5)	1.21 (0.93–1.58)	0.156	1.20 (0.92–1.57)	0.186	
High PA + Long SD	269/941 (28.6)	562/1887 (29.8)	1.12 (0.89–1.41)	0.342	1.11 (0.87–1.41)	0.403	

Abbreviations: CCI, Charlson comorbidity index; Low PA, low-intensity physical activity; High PA, moderate- to high-intensity physical activity; OR, odds ratio; PA, physical activity; SD, sunshine duration. * Conditional logistic regression for subgroup analyses according to income, and region of residence, un-conditional logistic regression for subgroup analyses according to obesity, smoking, and alcohol consumption, significance at < 0.05 with Bonferroni correction ($\alpha = 0.05/3$). † In subgroup analyses according to income and region of residence, models were stratified by age, sex, income, and region of residence. In subgroup analyses according to obesity, smoking, and alcohol consumption were not stratified. ‡ For conditional logistic regression, the adjusted model was adjusted for obesity, smoking, alcohol consumption, osteoporosis, and CCI scores, and for the un-conditional logistic regression, the adjusted model was adjusted for above variables plus age, sex, income, and region of residence.

Table S4. Odds ratios (95% confidence intervals) of moderate- to high-intensity physical activity for vertebral fracture and of long sunshine duration for vertebral fracture with subgroup analyses according to age, sex, income, region of residence, sunshine duration (or physical activity) status of obesity, smoking, and alcohol consumption, and osteoporosis history.

Characteristics	Vertebral fracture	Control I	OR (95% confidence intervals) for Vertebral Fracture				*P* value for Interaction
	(exposure/total, %)	(exposure/total, %)	Crude†	*P* value	Adjusted†‡	*P* value	
OR of High PA (reference: Low PA) for vertebral fracture							
Total participants (*n* =20,574)	2547/6858 (37.1)	5680/13716 (41.4)	0.83 (0.78–0.89)	<0.001*	0.85 (0.80–0.90)	<0.001*	
Age < 60 years old (*n* = 6300)	871/2100 (41.5)	1908/4200 (45.4)	0.85 (0.77–0.95)	0.003*	0.86 (0.78–0.96)	0.008*	0.362
Age ≥ 60 years old (*n* = 14,274)	1676/4758 (35.2)	3772/9516 (39.6)	0.83 (0.77–0.89)	<0.001*	0.84 (0.78–0.90)	<0.001*	
Males (*n* = 3207)	434/1069 (40.6)	1031/2138 (48.2)	0.73 (0.63–0.85)	<0.001*	0.77 (0.66–0.90)	0.001*	0.150
Females (*n* = 17,367)	2113/5789 (36.5)	4649/11,578 (40.2)	0.85 (0.80–0.91)	<0.001*	0.86 (0.81–0.92)	<0.001*	
Low income (*n* = 9093)	1050/3031 (34.6)	2416/6062 (39.9)	0.80 (0.73–0.87)	<0.001*	0.80 (0.73–0.88)	<0.001*	0.332
High income (*n* = 11,481)	1497/3827 (39.1)	3264/7654 (42.6)	0.86 (0.80–0.93)	<0.001*	0.88 (0.81–0.96)	0.002*	
Urban (*n* = 6321)	931/2107 (44.2)	2041/4214 (48.4)	0.84 (0.76–0.94)	0.001*	0.86 (0.77–0.95)	0.005*	0.871
Rural (*n* = 14,253)	1616/4751 (34.0)	3639/9502 (38.3)	0.83 (0.77–0.89)	<0.001*	0.84 (0.78–0.91)	<0.001*	
Short SD (*n* = 10,426)	1198/3514 (34.1)	2680/6912 (38.8)	0.82 (0.75–0.89)	<0.001*	0.83 (0.76–0.90)	<0.001*	0.549
Long SD (*n* = 10,148)	1,349/3,344 (40.3)	3,000/6,804 (44.1)	0.86 (0.79–0.93)	<0.001*	0.87 (0.80–0.94)	0.001*	
Underweight (*n* = 713)	95/295 (32.2)	150/418 (35.9)	0.85 (0.62–1.16)	0.259	0.85 (0.62–1.18)	0.333	0.270
Normal weight (*n* = 7358)	935/2543 (36.8)	2018/4815 (41.9)	0.81 (0.73–0.89)	<0.001*	0.83 (0.75–0.92)	<0.001*	
Overweight (*n* = 5,250)	651/1,98 (38.3)	1526/3552 (43.0)	0.83 (0.73–0.93)	0.002*	0.84 (0.75–0.95)	0.005*	
Obese (*n* = 7253)	866/2322 (37.3)	1986/4931 (40.3)	0.88 (0.80–0.98)	0.004*	0.87 (0.79–0.97)	0.008*	
Nonsmoker (*n* = 18,448)	2268/6128 (37.0)	5022/12,320 (40.8)	0.85 (0.80–0.91)	<0.001*	0.86 (0.81–0.92)	<0.001*	0.197
Past and current smoker (*n* = 2126)	279/730 (38.2)	658/1396 (47.1)	0.69 (0.58–0.83)	<0.001*	0.71 (0.59–0.86)	<0.001*	
Alcohol consumption < 1 time a week (*n* = 15,318)	1846/5129 (36.0)	4129/10,189 (40.5)	0.83 (0.77–0.89)	<0.001*	0.84 (0.78–0.90)	<0.001*	0.406
Alcohol consumption ≥ 1 time a week (*n* = 5256)	701/1,729 (40.5)	1551/3527 (44.0)	0.87 (0.77–0.98)	0.009*	0.87 (0.78–0.98)	0.027	
Nonosteoporosis (*n* = 3492)	372/997 (37.3)	1107/2495 (44.4)	0.75 (0.64–0.87)	<0.001*	0.73 (0.63–0.85)	<0.001*	0.062
Osteoporosis (*n* = 17,082)	2175/5861 (37.1)	4573/11,221 (40.8)	0.86 (0.80–0.92)	<0.001*	0.87 (0.81–0.93)	<0.001*	
OR of Long SD (reference: Short SD) for vertebral fracture							
Total participants (*n* =20,574)	3344/6858 (48.8)	6,804/13,716 (49.6)	0.96 (0.91–1.02)	0.229	0.99 (0.93–1.05)	0.719	
Age < 60 years old (*n* = 6300)	1032/2100 (49.1)	2167/4200 (51.6)	0.90 (0.80–1.00)	0.054	0.90 (0.81–1.01)	0.072	0.092
Age ≥ 60 years old (*n* = 14,274)	2312/4758 (48.6)	4637/9516 (48.7)	0.99 (0.92–1.07)	0.872	1.03 (0.96–1.11)	0.419	
Males (*n* = 3207)	503/1069 (47.1)	1029/2138 (48.1)	0.95 (0.81–1.11)	0.541	0.99 (0.84–1.16)	0.876	0.874
Females (*n* = 17,367)	2841/5789 (49.1)	5775/11,578 (49.9)	0.97 (0.90–1.03)	0.295	0.99 (0.92–1.06)	0.741	
Low income (*n* = 9093)	1427/3031 (47.1)	2912/6062 (48.0)	0.96 (0.87–1.05)	0.362	0.98 (0.89–1.08)	0.665	0.694
High income (*n* = 11,481)	1917/3827 (50.1)	3892/7654 (50.9)	0.97 (0.89–1.05)	0.424	1.00 (0.92–1.08)	0.915	
Urban (*n* = 6321)	1487/2107 (70.6)	2980/4214 (70.7)	0.99 (0.88–1.12)	0.906	1.00 (0.89–1.13)	0.957	0.574

Rural (*n* = 14,253)	1857/4751 (39.1)	3824/9502 (40.2)	0.95 (0.89–1.02)	0.181	0.98 (0.91–1.06)	0.622	
Low PA (*n* = 12,347)	1995/4311 (46.3)	3804/8036 (47.3)	0.96 (0.89–1.03)	0.210	0.98 (0.90–1.06)	0.573	0.549
High PA (*n* = 8227)	1349/2547 (53.0)	3000/5680 (52.8)	1.01 (0.92–1.11)	0.997	1.01 (0.91–1.11)	0.869	
Underweight (*n* = 713)	137/295 (46.4)	202/418 (48.3)	0.93 (0.69–1.25)	0.639	1.00 (0.72–1.38)	0.979	0.089
Normal weight (*n* = 7358)	1216/2543 (47.8)	2430/4815 (50.5)	0.90 (0.82–0.99)	0.071	0.94 (0.85–1.04)	0.259	
Overweight (*n* = 5250)	846/1698 (49.8)	1778/3552 (50.1)	0.99 (0.88–1.11)	0.926	1.01 (0.89–1.14)	0.884	
Obese (*n* = 7253)	1145/2322 (49.3)	2394/4,931 (48.6)	1.03 (0.93–1.14)	0.952	1.03 (0.93–1.15)	0.530	
Nonsmoker (*n* = 18,448)	2994/6128 (48.9)	6104/12,320 (49.6)	0.97 (0.92–1.03)	0.330	0.99 (0.93–1.06)	0.811	0.718
Past and current smoker (*n* = 2126)	350/730 (48.0)	700/1396 (50.1)	0.92 (0.77–1.10)	0.381	0.96 (0.79–1.16)	0.643	
Alcohol consumption < 1 time a week (*n* = 15,318)	2298/5129 (44.8)	4613/10,189 (45.3)	0.98 (0.92–1.05)	0.695	1.01 (0.94–1.09)	0.753	0.391
Alcohol consumption ≥ 1 time a week (*n* = 5256)	1046/1729 (60.5)	2191/3,527 (62.1)	0.93 (0.83–1.05)	0.105	0.91 (0.81–1.04)	0.161	
Nonosteoporosis (*n* = 3492)	483/997 (48.5)	1327/2495 (53.2)	0.83 (0.71–0.96)	0.005*	0.83 (0.71–0.97)	0.018	0.021
Osteoporosis (*n* = 17,082)	2861/5861 (48.8)	5477/11,221 (48.8)	1.00 (0.94–1.07)	0.926	1.02 (0.96–1.09)	0.523	

Abbreviations: CCI, Charlson comorbidity index; Low PA, low-intensity physical activity; High PA, moderate- to high-intensity physical activity; OR, odds ratio; PA, physical activity; SD, sunshine duration * Conditional logistic regression was used in analysis for total participants and subgroup analyses according to age, sex, income, and region of residence. Un-conditional logistic regression was used in subgroup analyses according to SD (or PA), status of obesity, smoking, alcohol consumption, and osteoporosis history, significance at P < 0.05 with Bonferroni correction (α = 0.05/3). † Models were stratified by age, sex, income, and region of residence in total participants, subgroup analyses according to age, sex, income, and region of residence. ‡ Adjusted model was adjusted for SD (or PA) obesity, smoking, alcohol consumption, CCI scores, and osteoporosis history for total participants and for subgroup analyses according to age, sex, income, and region of residence. In subgroup analyses according to SD (or PA), status of obesity, smoking, alcohol consumption, and osteoporosis history, adjusted model was adjusted for above variables plus age, sex, income, and region of residence.

Table S5. Odds ratios (95% confidence intervals) of moderate- to high-intensity physical activity for hip fracture and of long sunshine duration for hip fracture with subgroup analyses according to age, sex, income, region of residence, sunshine duration (or physical activity) status of obesity, smoking, and alcohol consumption, and osteoporosis history.

**Characteristics**	**Hip fracture**	**Control II**	**OR (95% confidence intervals) for Hip Fracture**				***P* value for Interaction**
	**(exposure/total, %)**	**(exposure/total, %)**	**Crude†**	***P* value**	**Adjusted†‡**	***P* value**	
OR of High PA (reference: Low PA) for hip fracture							
Total participants (*n* = 3288)	386/1096 (35.2)	870/2192 (39.7)	0.82 (0.71–0.96)	0.012*	0.83 (0.71–0.97)	0.020	
Age < 60 years old (*n* = 585)	81/195 (41.5)	189/390 (48.5)	0.76 (0.54–1.07)	0.116	0.70 (0.49–1.02)	0.065	0.587
Age ≥ 60 years old (*n* = 2703)	305/901 (33.9)	681/1802 (37.8)	0.84 (0.71–1.00)	0.044	0.85 (0.71–1.01)	0.064	
Males (*n* = 873)	115/291 (39.5)	276/582 (47.4)	0.73 (0.55–0.97)	0.029	0.70 (0.52–0.94)	0.019	0.196
Females (*n* = 2415)	271/805 (33.7)	594/1610 (36.9)	0.87 (0.72–1.04)	0.115	0.89 (0.74–1.07)	0.199	
Low income (*n* = 1476)	171/492 (34.8)	370/984 (37.6)	0.88 (0.70–1.11)	0.279	0.88 (0.69–1.12)	0.304	0.905
High income (*n* = 1812)	215/604 (35.6)	500/1208 (41.4)	0.78 (0.64–0.96)	0.017	0.78 (0.64–0.97)	0.023	
Urban (*n* = 1068)	147/356 (41.3)	336/712 (47.2)	0.79 (0.61–1.02)	0.070	0.80 (0.62–1.05)	0.102	0.680
Rural (*n* = 2220)	239/740 (32.3)	534/1480 (36.1)	0.84 (0.70–1.02)	0.076	0.85 (0.70–1.03)	0.100	
Short SD (*n* = 1584)	164/511 (32.1)	392/1073 (36.5)	0.82 (0.66–1.03)	0.094	0.82 (0.65–1.04)	0.104	0.982

Long SD (*n* = 1704)	222/585 (38.0)	478/1119 (42.7)	0.82 (0.67–1.01)	0.051	0.83 (0.67–1.02)	0.081	
Underweight (*n* = 183)	38/98 (38.8)	28/85 (32.9)	1.29 (0.70–2.37)	0.854	1.21 (0.58–2.53)	0.609	0.626
Normal weight (*n* = 1242)	144/422 (34.1)	340/820 (41.5)	0.73 (0.57–0.93)	0.010*	0.72 (0.56–0.93)	0.012*	
Overweight (*n* = 830)	85/250 (34.0)	239/580 (41.2)	0.74 (0.54–1.00)	0.072	0.76 (0.55–1.05)	0.101	
Obese (*n* = 1033)	119/326 (36.5)	263/707 (37.2)	0.97 (0.74–1.27)	0.841	0.98 (0.74–1.31)	0.906	
Nonsmoker (*n* = 2738)	309/892 (34.6)	707/1846 (38.3)	0.85 (0.72–1.01)	0.057	0.86 (0.72–1.02)	0.083	0.640
Past and current smoker (*n* = 550)	77/204 (37.8)	163/346 (47.1)	0.68 (0.48–0.97)	0.050	0.71 (0.48–1.03)	0.070	
Alcohol consumption < 1 time a week (*n* = 2366)	275/825 (33.3)	604/1541 (39.2)	0.78 (0.65–0.93)	0.003*	0.76 (0.63–0.92)	0.004*	0.111
Alcohol consumption ≥ 1 time a week (*n* = 922)	111/271 (41.0)	266/651 (40.9)	1.00 (0.75–1.34)	0.945	1.03 (0.76–1.40)	0.854	
Nonosteoporosis (*n* = 691)	79/237 (33.3)	192/454 (42.3)	0.68 (0.49–0.95)	0.029	0.64 (0.45–0.92)	0.014*	0.104
Osteoporosis (*n* = 2597)	307/859 (35.7)	678/1738 (39.0)	0.87 (0.73–1.03)	0.083	0.88 (0.73–1.04)	0.139	
OR of Long SD (reference: Short SD) for hip fracture							
Total participants (*n* = 3288)	585/1096 (53.4)	1119/2,192 (51.1)	1.11 (0.95–1.30)	0.180	1.17 (1.00–1.37)	0.057	
Age < 60 years old (*n* = 585)	109/195 (55.9)	201/390 (51.5)	1.25 (0.85–1.83)	0.266	1.16 (0.76–1.77)	0.488	0.784
Age ≥ 60 years old (*n* = 2703)	476/901 (52.8)	918/1802 (50.9)	1.09 (0.92–1.29)	0.330	1.17 (0.98–1.39)	0.086	
Males (*n* = 873)	167/291 (57.4)	300/582 (51.6)	1.33 (0.97–1.81)	0.074	1.30 (0.93–1.81)	0.124	0.568
Females (*n* = 2415)	418/805 (51.9)	819/1610 (50.9)	1.05 (0.88–1.25)	0.606	1.13 (0.94–1.37)	0.184	
Low income (*n* = 1476)	252/492 (51.2)	485/984 (49.3)	1.10 (0.87–1.38)	0.449	1.15 (0.90–1.47)	0.270	0.754
High income (*n* = 1812)	333/604 (55.1)	634/1208 (52.5)	1.12 (0.92–1.38)	0.264	1.18 (0.95–1.46)	0.128	
Urban (*n* = 1068)	244/356 (68.5)	492/712 (69.1)	0.97 (0.73–1.30)	0.843	1.05 (0.78–1.43)	0.744	0.329
Rural (*n* = 2220)	341/740 (46.1)	627/1480 (42.4)	1.17 (0.98–1.41)	0.088	1.23 (1.01–1.48)	0.036	
Low PA (*n* = 2032)	363/710 (51.1)	641/1322 (48.5)	1.11 (0.93–1.33)	0.321	1.17 (0.96–1.42)	0.119	0.982
High PA (*n* = 1256)	222/386 (57.5)	478/870 (54.9)	1.11 (0.87–1.41)	0.340	1.14 (0.88–1.48)	0.315	
Underweight (*n* = 183)	53/98 (54.1)	42/85 (49.4)	1.21 (0.67–2.16)	0.796	1.27 (0.64–2.51)	0.493	0.663
Normal weight (*n* = 1242)	228/422 (54.0)	415/820 (50.6)	1.15 (0.91–1.45)	0.278	1.20 (0.93–1.54)	0.160	
Overweight (*n* = 830)	130/250 (52.0)	296/580 (51.0)	1.04 (0.77–1.40)	0.832	1.13 (0.82–1.56)	0.454	
Obese (*n* = 1033)	174/326 (53.4)	366/707 (51.8)	1.07 (0.82–1.39)	0.596	1.13 (0.85–1.49)	0.404	
Nonsmoker (*n* = 2738)	473/892 (53.0)	951/1846 (51.5)	1.06 (0.91–1.25)	0.437	1.12 (0.94–1.33)	0.198	0.150
Past and current smoker (*n* = 550)	112/204 (54.9)	168/346 (48.6)	1.29 (0.91–1.83)	0.091	1.49 (1.01–2.19)	0.045	
Alcohol consumption < 1 time a week (*n* = 2366)	418/825 (50.7)	732/1541 (47.5)	1.14 (0.96–1.34)	0.129	1.19 (0.99–1.42)	0.064	0.694
Alcohol consumption ≥ 1 time a week (*n* = 922)	167/271 (61.6)	387/651 (59.5)	1.10 (0.82–1.47)	0.454	1.11 (0.81–1.51)	0.527	
Nonosteoporosis (*n* = 691)	132/237 (55.7)	241/454 (53.1)	1.11 (0.81–1.52)	0.334	1.22 (0.85–1.75)	0.271	0.721
Osteoporosis (*n* = 2597)	453/859 (52.7)	878/1738 (50.5)	1.09 (0.93–1.29)	0.327	1.15 (0.97–1.38)	0.107	

Abbreviations: CCI, Charlson comorbidity index; Low PA, low-intensity physical activity; High PA, moderate- to high-intensity physical activity; OR, odds ratio; PA, physical activity; SD, sunshine duration * Conditional logistic regression was used in analysis for total participants and subgroup analyses according to age, sex, income, and region of residence.

Un-conditional logistic regression was used in subgroup analyses according to SD (or PA), status of obesity, smoking, alcohol consumption, and osteoporosis history, significance at P < 0.05 with Bonferroni correction (α = 0.05/3). † Models were stratified by age, sex, income, and region of residence in total participants, subgroup analyses according to age, sex, income, and region of residence. ‡ Adjusted model was adjusted for SD (or PA) obesity, smoking, alcohol consumption, CCI scores, and osteoporosis history for total participants and for subgroup analyses according to age, sex, income, and region of residence. In subgroup analyses according to SD (or PA), status of obesity, smoking, alcohol consumption, and osteoporosis history, adjusted model was adjusted for above variables plus age, sex, income, and region of residence.

Table S6. Odds ratios (95% confidence intervals) of moderate- to high-intensity physical activity for distal radius fracture and of long sunshine duration for distal radius fracture with subgroup analyses according to age, sex, income, region of residence, sunshine duration (or physical activity) status of obesity, smoking, and alcohol consumption, and osteoporosis history.

Characteristics	Distal radius fracture (exposure/total, %)	Control III (exposure/total, %)	OR (95% confidence intervals) for Distal Radius Fracture				*P* value for Interaction
			Crude†	*P* value	Adjusted†‡	*P* value	
Odds ratio of High PA (reference: Low PA) for distal radius fracture							
Total participants (*n* = 12,447)	1819/4149 (43.8)	3547/8298 (42.8)	1.05 (0.97–1.13)	0.241	1.05 (0.97–1.13)	0.230	
Age < 60 years old (*n* = 7428)	1136/2476 (45.9)	2236/4952 (45.2)	1.03 (0.94–1.14)	0.552	1.03 (0.93–1.13)	0.592	0.896
Age ≥ 60 years old (*n* = 5019)	683/1673 (40.8)	1311/3346 (39.2)	1.07 (0.95–1.21)	0.256	1.09 (0.96–1.23)	0.189	
Males (*n* = 723)	101/241 (41.9)	231/482 (47.9)	0.78 (0.57–1.07)	0.127	0.80 (0.58–1.10)	0.162	0.073
Females (*n* = 11,724)	1718/3908 (44.0)	3316/7816 (42.4)	1.07 (0.99–1.15)	0.111	1.07 (0.99–1.15)	0.110	
Low income (*n* = 5601)	792/1867 (42.4)	1571/3734 (42.1)	1.02 (0.91–1.14)	0.803	1.02 (0.91–1.14)	0.731	0.915
High income (*n* = 6846)	1027/2282 (45.0)	1976/4564 (43.3)	1.07 (0.97–1.19)	0.175	1.07 (0.97–1.19)	0.191	
Urban (*n* = 4863)	813/1621 (50.2)	1533/3242 (47.3)	1.12 (1.00–1.27)	0.059	1.12 (0.99–1.26)	0.062	0.137
Rural (*n* = 7584)	1006/2528 (39.8)	2014/5056 (39.8)	1.00 (0.91–1.10)	0.973	1.00 (0.91–1.11)	0.971	
Short SD (*n* = 6197)	878/2074 (42.3)	1685/4123 (40.9)	1.06 (0.96–1.18)	0.245	1.08 (0.97–1.20)	0.189	0.673
Long SD (*n* = 6250)	941/2075 (45.4)	1862/4175 (44.6)	1.03 (0.93–1.15)	0.586	1.03 (0.93–1.14)	0.605	
Underweight (*n* = 335)	43/124 (34.7)	78/211 (37.0)	0.91 (057–1.44)	0.554	0.86 (0.52–1.41)	0.548	0.851
Normal weight (*n* = 4513)	716/1600 (44.8)	1253/2913 (43.0)	1.07 (0.94–1.21)	0.233	1.08 (0.95–1.22)	0.223	
Overweight (*n* = 3268)	488/1111 (43.9)	953/2157 (44.2)	0.99 (0.86–1.15)	0.831	0.99 (0.85–1.15)	0.890	
Obese (*n* = 4331)	572/1314 (43.5)	1263/3017 (41.9)	1.07 (0.94–1.22)	0.300	1.08 (0.95–1.23)	0.263	
Nonsmoker (*n* = 11,782)	1725/3925 (44.0)	3335/7857 (42.5)	1.06 (0.98–1.15)	0.112	1.07 (0.99–1.15)	0.099	0.252
Past and current smoker (*n* = 665)	94/224 (42.0)	212/441 (48.1)	0.78 (0.57–1.08)	0.132	0.78 (0.56–1.08)	0.135	
Alcohol consumption < 1 time a week (*n* = 9619)	1398/3208 (43.6)	2692/6411 (42.0)	1.07 (0.98–1.16)	0.119	1.08 (0.99–1.17)	0.098	0.324
Alcohol consumption ≥ 1 time a week (*n* = 2828)	421/941 (44.7)	855/1887 (45.3)	0.98 (0.84–1.14)	0.719	0.97 (0.83–1.14)	0.728	
Nonosteoporosis (*n* = 2247)	319/760 (42.0)	661/1487 (44.5)	0.90 (0.76–1.08)	0.276	0.92 (0.77–1.09)	0.330	0.074
Osteoporosis (*n* = 10,200)	1500/3389 (44.3)	2886/6811 (42.4)	1.08 (0.99–1.17)	0.070	1.08 (1.00–1.18)	0.060	
Odds ratio of Long SD (reference: Short SD) for distal radius fracture							
Total participants (*n* = 12,447)	2075/4149 (50.0)	4175/8298 (50.3)	0.99 (0.91–1.07)	0.738	0.98 (0.91–1.07)	0.671	
Age < 60 years old (*n* = 7428)	1261/2476 (50.9)	2483/4952 (50.1)	1.04 (0.94–1.15)	0.501	1.02 (0.92–1.14)	0.654	0.304
Age ≥ 60 years old (*n* = 5019)	814/1673 (48.7)	1692/3346 (50.6)	0.92 (0.81–1.04)	0.174	0.92 (0.81–1.05)	0.205	

Males (*n* = 723)	125/241 (51.9)	262/482 (54.4)	0.88 (0.62–1.25)	0.476	0.88 (0.62–1.25)	0.478	0.545
Females (*n* = 11,724)	1950/3908 (49.9)	3913/7816 (50.1)	0.99 (0.92–1.08)	0.858	0.99 (0.91–1.07)	0.722	
Low income (*n* = 5601)	882/1867 (47.2)	1815/3734 (48.6)	0.94 (0.84–1.06)	0.311	0.95 (0.84–1.07)	0.400	0.538
High income (*n* = 6846)	1,193/2,282 (52.3)	2360/4564 (51.7)	1.03 (0.92–1.14)	0.638	1.02 (0.91–1.13)	0.789	
Urban (*n* = 4863)	1,110/1,621 (68.5)	2234/3242 (68.9)	0.98 (0.86–1.12)	0.756	0.97 (0.85–1.11)	0.652	0.836
Rural (*n* = 7584)	965/2,528 (38.2)	1941/5056 (38.4)	0.99 (0.90–1.09)	0.854	1.00 (0.90–1.10)	0.926	
Low PA (*n* = 7081)	1,134/2,330 (48.7)	2313/4751 (48.7)	1.00 (0.91–1.10)	0.758	1.01 (0.91–1.13)	0.830	0.673
High PA (*n* = 5366)	941/1,819 (51.7)	1862/3547 (52.5)	0.97 (0.87–1.09)	0.392	0.95 (0.84–1.07)	0.418	
Underweight (*n* = 335)	55/124 (44.4)	119/211 (56.4)	0.62 (0.39–0.96)	0.083	0.67 (0.41–1.10)	0.114	0.365
Normal weight (*n* = 4513)	838/1600 (52.4)	1470/2913 (50.5)	1.08 (0.96–1.22)	0.157	1.09 (0.96–1.25)	0.173	
Overweight (*n* = 3268)	555/1111 (50.0)	1066/2157 (49.4)	1.02 (0.88–1.18)	0.753	0.97 (0.83–1.13)	0.664	
Obese (*n* = 4331)	627/1314 (47.7)	1520/3017 (50.4)	0.90 (0.79–1.02)	0.221	0.92 (0.80–1.06)	0.250	
Nonsmoker (*n* = 11,782)	1951/3925 (49.7)	3940/7857 (50.2)	0.98 (0.91–1.06)	0.700	0.98 (0.90–1.06)	0.640	0.450
Past and current smoker (*n* = 665)	124/224 (55.4)	235/441 (53.3)	1.09 (0.79–1.50)	0.921	1.02 (0.72–1.45)	0.921	
Alcohol consumption < 1 time a week (*n* = 9619)	1455/3208 (45.4)	2976/6411 (46.4)	0.96 (0.88–1.04)	0.365	0.96 (0.87–1.05)	0.324	0.109
Alcohol consumption ≥ 1 time a week (*n* = 2828)	620/941 (65.9)	1199/1887 (63.5)	1.11 (0.94–1.31)	0.304	1.10 (0.93–1.31)	0.267	
Nonosteoporosis (*n* = 2247)	382/760 (50.3)	807/1487 (54.3)	0.85 (0.72–1.01)	0.075	0.86 (0.71–1.04)	0.110	0.057
Osteoporosis (*n* = 10,200)	1693/3389 (50.0)	3368/6811 (49.5)	1.02 (0.94–1.11)	0.645	1.02 (0.93–1.11)	0.738	

Abbreviations: CCI, Charlson comorbidity index; Low PA, low-intensity physical activity; High PA, moderate- to high-intensity physical activity; OR, odds ratio; PA, physical activity; SD, sunshine duration * Conditional logistic regression was used in analysis for total participants and subgroup analyses according to age, sex, income, and region of residence. Un-conditional logistic regression was used in subgroup analyses according to SD (or PA), status of obesity, smoking, alcohol consumption, and osteoporosis history, significance at $P < 0.05$ with Bonferroni correction ($\alpha = 0.05/3$). † Models were stratified by age, sex, income, and region of residence in total participants, subgroup analyses according to age, sex, income, and region of residence. ‡ Adjusted model was adjusted for SD (or PA) obesity, smoking, alcohol consumption, CCI scores, and osteoporosis history for total participants and for subgroup analyses according to age, sex, income, and region of residence. In subgroup analyses according to SD (or PA), status of obesity, smoking, alcohol consumption, and osteoporosis history, adjusted model was adjusted for above variables plus age, sex, income, and region of residence.
